# Comparative Physiology of Menstruation

**Published:** 1884-04

**Authors:** Alfred Wiltshire

**Affiliations:** Lond.


### THE COMPARATIVE PHYSIOLOGY OF MENSTRUATION.

BY ALFRED WILTSHIRE, M.D., F.R.C.P., LOND.

[From the London Veterinary Journal.]

Continued from Page 65.

ANALOGOUS susceptibility is displayed by the human female in the disturbance, mostly arrest, of menstruation that so often attends change of residence. I have met with innumerable instances of this. Nothing is more common than for maid-servants coming from the country to London to have amenorrhœa for some months. Among ladies, many instances of disorder have come under my notice from foreign travel; but the effects are not always injurious. Making due allowances, then, we may now review the evidence at our disposal.

Mr. Simpson's statements, based upon observations made on his own cattle, are as explicit as they are reliable. He states that his heifers usually arrive at puberty at from six to nine months, and sometimes even earlier; and that, after the establishment of the symptoms of heat, or "bulling," the rut recurs, when the bull is not allowed access, with strict regularity every twenty-one days, or three weeks. Œstro-menstruation is shown by swelling of the vulva, which at first weeps an odorous mucus, but soon this becomes quite red from the presence of blood; but when that stage is attained, the heat rapidly subsides, intercourse being then refused. Mr. West, Mr. Simpson's excellent veterinary surgeon, emphatically confirms these observations, and adduces his very large acquaintance with analogous phenomena, personally observed, in other animals, as the mare, female ass, bitch, cat, etc., as well as in cows.

Mr. West made a statement to me which is amply corroborated by remarks of Mr. Darwin's, namely, that in the rougher Welsh and Highland cattle, which lead harder lives, maturity, or the epoch of puberty, arrives much later than in Alderneys or shorthorns; but he adds that a flux of sanguineous character equally occurs in the females of those breeds at the time of the rut. I am indebted to Mr. West for information respecting a remarkable case of "impervious vagina in a heifer," which occurred in his practice, and which illustrates the occurrence of sanguineous menstruation in the heifer. Mr. West was called to see a heifer supposed to be in labor, and unable to calve. On examination, he found the vagina to end in an impervious canal three inches from the vulva. Thinking the fœtus must be dead and abortion prevented by the state of the vagina, Mr. West advised that nothing should be done, hoping that the pains might subside. They continued, however, and the heifer lost flesh. Four months afterwards, Mr. West determined to cut through the obstruction. This he did, but having passed it, he found the os uteri contracted; accordingly, into this he inserted a whalebone sound, and dilated it a little, when a reddish-brown offensive fluid began to escape. About two quarts altogether came away, but no signs of a fœtus. The beast recovered. Mr. West concludes with the remark that the fluid "should have come away as a periodic discharge, but was prevented by the impervious state of the vagina, which must have also rendered impregnation impossible."

Mr. West was also so good as to refer me to a similar case published by Mr. Macgillivray, in Vol. III. of the *Veterinary Journal*, p. 443. In this case there was an impervious vagina in a heifer, which had never been put to the bull. The creature had severe bearing-down pains, and was thought to have obstruction of the bowels. A passage was forced into the generative canal, and dark-brown offensive fluid escaped. Mr. Macgillivray regarded the case as proving "the existence of a menstrual discharge in the brute female, analogous to that in the human female." He considered that the "œstral" product had never found vent from the uterus. These views were combated by another veterinary surgeon, Mr. Gerrard, but his arguments, though plausibly advanced, were quite inconclusive. Mr. Gerrard points out that adhesion of the vagina sometimes arises from injury done in copulation; but admitting this as a possible though rare occurrence, it does not apply in Mr. Macgillivray's case. Nor is Mr. Gerrard's argument on the "sero-sanguineous" nature of the fluid valid; for, as will be shown, the sanguineous character of the fluid diminishes as we descend in the organic scale; even in women the catamenial discharge is sometimes abundantly mucous. Mr. Macgillivray replies to his opponent's strictures in the same volume (vol. iv.), and conclusively shows that his case was one of impervious vagina. He goes on to express the very decided conviction that the lower animals do certainly have an "œstral" discharge resembling the catamenia of women, though it is not so invariable, copious, or well-defined as in women. He then gives instances of excessive flow in the cow and mare, and concludes by saying: "The discharge of a more or less quantity of a blood-like fluid in heifers and cows during the cessation of 'heat,' is so common, and so well known to all herdsmen, as scarcely to require any notice here."

Mr. Gerrard, admitting that the "œstral" discharge is frequently more or less tinged with blood in the lower animals, considers that a sanguineous flow is the exception and not the rule ; but it is obvious that he is opposed to authorities like Fleming and Saint Cyr, as well as to the observation of most competent inquirers. Probably, when it is understood that, in accordance with the law of evolution, the discharge in the lower females is normally less sanguineous than in women, these discrepancies between undoubtedly honest observers will disappear.

Laycock (*Nervous Diseases of Women*, p. 42) says:—"The menstrual period has been considered analogous to the heat of lower animals by numerous writers. Reaumur and others down to Cruikshank and Blundell, have described the state of the organs of generation in brute females during this period ; they have been found fuller than usual of blood, the fallopian tubes in a state of excitement, or applied to the ovary, the latter enlarged and studded with developed Graafian vesicles, and a serous blood-colored fluid discharged from the vagina. There are various considerations which serve to support this opinion."

"The following may be considered as the true state of the case respecting menstruation. Since the uterus itself is not an essential organ of generation, but merely superadded, and since the influence of the ovaria and testes over all the other processes and organs connected with generation, including the existence of the uterus and its development during gestation, has been demonstrated, there appears not the slightest reason for withdrawing the phenomena of menstruation from their agency. It is in the ovaria, then, that we have to look for the causes of this process. There is every reason to believe that Graafian vesicles are coming forward at intervals during the whole period in which the reproductive organs are active; that these vesicles enlarge and burst in succession, and shed the contained ovula, whether sexual connection takes place or not; and that, from recent researches, these changes in them take place at each menstrual nisus. If we remember that, during the period of heat in the lower mammals, as the ewe and the sow, and of spawning and egg-laying in birds, fishes, reptiles, insects—indeed, of every class of oviparous animals—these ovula become developed, and are shed, whether they be fructified or not, recurring at the same time to previous statements, we cannot help coming to the conclusion that the period of menstruation is precisely analogous to the period of heat; that there is, in fact, an excited state of the ovaria at each period when the ovula are shed; and that the capability of performing this periodic function distinguishes the ovaria of the woman from those of the impubescent girl and virago. If, at this period, an ovulum be vivified by the male semen, conception takes place; and this hypothesis at once explains the doctrine that women more readily conceive at the menstrual period maintained by Hippocrates, Galen, and their numerous copyists among the ancients, by Dr. Montgomery and others in later times, and generally believed by females themselves. When conception has taken place, a new action is set up in the ovaria; which may be considered as a permanent stimulus to the whole of the generative organs; and although the usual nisus may and does occur during pregnancy, its effects are rendered less obvious from its permanency.* If, on the other hand, the discharged ovulum or ovula be unimpregnated, the same process is repeated at the next menstrual period, and so continues until age, disease, or conception interferes with the ovarian system. But we shall find that this periodic movement is not limited to the ovaria, but that it is an affection of the general system in which the ovaria partake; and that it is through these the secondary system in connection with them

* Fleming (*Vet. Obstetrics*, p. 52) says: "Franck has convinced himself, by *post mortem* examination of mares, of the possibility of ova being thrown off from the ovary during pregnancy."

is influenced, and all the attendant phenomena (those of menstruation excited."

"Recurring to our previous statements, it appears that in many animals the development of the testes and ovaria, and the shedding of the ovula and spermatic fluid, occur at definite seasons of the year, and, for the most part, in spring and autumn. The heat also of those animals, in which the genitals are in continuous activity, occurs at fixed periods, and these must be compared with the periodic movement of the human female. Again, the period of gestation in women is a multiple of the menstrual period, and it will be useful to inquire into the relations of the periods of utero-gestation in animals generally to that of woman."

"Are the lower animals influenced by a periodic nisus, weekly or monthly? This question I shall attempt to answer in the affirmative, as well as my limited space will allow. It has already been remarked that the change from fœtal to uterine [? extra-uterine] life is a phase in development which in man occurs at the end of the tenth menstrual period of the female. This is correct as regards the general fact; but it must be added that slight labor pains occur at every menstrual period, but most particularly in the third, fifth, and seventh months of gestation; a fœtus of the last-mentioned age being able to maintain an independent existence. The period of incubation of the egg is strictly analogous to the periods of utero-gestation in mammalia; and the same remark is applicable to that of the ova of fishes, reptiles and insects, with due limitations. For example, in insects, the egg, larva, and chrysalis states correspond to the whole period between conception and puberty in mammalia. Mr. Kirby remarks that winged insects, many brachiopod crustacea, and the batrachian reptiles, in leaving the egg, only quit their first integument, answering to the chorion or external envelope of the human fœtus; they, therefore, still continue in the fœtal state." Laycock also quotes from the *Zoonomia* of the elder Darwin (Erasmus) that, "in mares and bitches, if the venereal orgasm be disappointed of its object, it recurs at monthly periods" (p. 60).

Laycock adds many illustrations of the periodical recurrence of heat at brief intervals in the lower animals (pp. 60-61); and mentions an instance of a cow which, while in calf, was in heat every three weeks, and calved three weeks after the last time of heat. Kolbe and Buffon are quoted with reference to menstruation in monkeys, the latter asserting that the following monkeys menstruate (besides the ourang-outang) :—" The Barbary ape, the ribbed-nose baboon, the lion-tailed baboon, the pig-tailed baboon, the hare-lip monkey, the Malbrouck (*simia sinica*), the white eyelid, the varied, the green, and the moustache monkey."

Laycock states that the rutting of the males is somewhat analogous to the heat of the females in respect of its periodicity, and remarks that "the ring-pigeon lays eggs for fourteen days after pairing, sits other fourteen days, and in fourteen more the young ones leave the nest. The goldfinch completes its nest in three days ; it is left unoccupied four days, when the first egg is laid. Reviewing the whole of the preceding facts, it appears a legitimate deduction that, in animals, changes occur in every three and a half, seven, fourteen, twenty-one, twenty-eight days, or at some definite number of weeks." In most of Laycock's statements I concur.

The valuable contributions of Pouchet to the subject of menstruation lend material support to the view that the function pervades the mammalian series, and is subordinate to the law of evolution already propounded. He remarks (*Théorie Positive de l'Ovulation Spontanée*, p. 201), that the commotion excited by the maturation of ovules, not only excites the genital apparatus, but reacts upon the whole individual. " Sometimes it is manifested but once during life ; the animal—suddenly exhausted by this concentration of all its vital forces—dies soon after having produced its ova ; this is observed in the majority of insects. The beings of more robust organization resist this act, and we see that during the middle period of their life they reproduce annually. The majority of fish, reptiles, amphibia, birds, and mammalia are in this case. There exists in these creatures a species of periodic growth, as G. Saint-Hilaire has said, during which the blood flows constantly towards the ovaries and excites an expansive movement." He then points out the heightening power of domestication, but insists that, even when the periods are rendered more frequent, they still show intermittance, which in woman is monthly. In the lower vertebrata, the sexual disturbance is not so conspicuous as in higher creatures. In some mammalia the genital orifices and adjacent parts show conspicuous excitement, often accompanied by sanguineous emissions. The names of Aristotle, Linnæus, Buffon, Cuvier, Blumenbach, Saint-Hilaire, and others are quoted in support.

Again: "If observation shows that ova are incontestably produced at fixed epochs in all invertebrate and vertebrate animals, since in them new generations appear constantly after regular and invariable periods, if, I say, that is admitted for all the zoological series, and it cannot even be contested as regards mammalia in a wild state, it becomes evident that the aberration observable in these latter that lived in our habitations, comes only from the new condition in which they are found; for attentive observation shows us that in them equally there are phases of excitation, and that it is during these that ovules are produced and that fecundation is possible. The condition of the human species enters entirely into this category, and if the periods when the reproduction is possible are very frequent in women, that belongs manifestly to the amenities of social life."

"In animals the flow of blood is ordinarily less abundant, and the period of excitement returns at longer intervals. Notwithstanding, there exist in the domain of mammology species which are nearly as much regular as certain women, and in which the flow appears almost as frequently." Dugès and Jourdan also remarked these analogies.

Isidore Geoffroy Saint-Hilaire says (Breschet, *Recherches Anatomiques et Physiologiques sur la Gestation des Quadrumanes*, Paris, 1845, p. 4):—"In monkeys the flow coincides in all the females with a swelling, more or less manifest, of the vulva and environing parts. This swelling, moderate in the females of the ape, is, on the contrary, very considerable in the females of many species of macacus, and in the species of cynocephalus. In all the latter it extends not only to the anus, but beyond, and it is so marked that all the orifice seems as if environed by a large collar. The skin at the same time becomes deeply red. In the mandrill, G. Cuvier compares, for volume, to a child's head, the unequal red and inflamed-looking protuberance which then forms around the anus. The same phenomena, though a little less pronounced, occur in the females of macacus; and even it often happens in these, for example, and in the females of rhesus and maimons, that the swelling extends to the inferior part of the tail, near the base." Again, Saint-Hilaire (*Histoire Naturelle des Mammiferes*, Paris, 1824) says:—"The females of apes, macacus, magots, cynocephalus, and probably of all other kinds of the first tribe, are subject to a flow periodically appearing from month to month. The matters emitted by the vulva are blood and mucosities, sometimes sanguinolent, sometimes white; the flow continues during six or eight days, and sometimes more. G. Cuvier fixed even at fifteen days the duration of the flow in a female mandrill which he made the subject of repeated observations."

Cuvier observed menstruation in carnivora, among others in the genets; and Lesson and Garnot recognized it in the flying-fox (pteropus), which Saint-Hilaire says is periodical. Haller quotes many authors who thought monkeys, cows, deer, and bitches offered evident traces of menstruation. Numann and Rainard made similar observations, while Pouchet had himself observed it in bitches, sows, cats, rabbits, and guinea-pigs, especially in the first. (Mr. Bartlett, the able superintendent of the Zoological Gardens, tells me that the discharge may be abundant in the bitch, and that the heat is sometimes long sustained in the carnivora). The wild pigeon produces only once or twice a year, while the varieties which it has given us through care, breed ten times a year, as Aristotle, Buffon, and Blumenbach have observed. Kuhlemann says sheep come to heat every fortnight; sows every fifteen to eighteen days. Kahleis and Numann, that cows come every month or three weeks; Greve, that mares come monthly; and Cuvier, that buffaloes, zebras, and monkeys, come also monthly. Courty also recognized the graduated relation of menstruation in the zoological series.

From his observations on sows, Pouchet concludes that the menstrual phenomena ordinarily precede the rupture of the Graafian follicles. P. 262:—"During menstruation the vagina of the sow offers a rosy tint, and the mucous fluid which it contains is slightly abundant. Microscopical examination showed me that the latter is composed of fragments of epithelium, cylindrical and pavement; mucous globules, and blood-corpuscles in small number. . . . The womb, before its bifurcation, is reddened, and its capillaries are highly injected with blood. . . . The cornua are excessively injected with blood. Their mucosa is considerably thickened and spongy, and of a deep red, and, in certain parts, the abundance of blood wherewith it is engorged gives it even a violet coloration. . . . Thus, then, menstruation in the sow is a demonstrated fact. As in the human species, there is an emission of blood ; but if this is not abundant, it is because this fluid is found in great part poured out in the immense extent of the internal generative apparatus." During the intermenstrual period, the vaginal and uterine mucous membranes are pale. Pouchet says that the appearances are absolutely similar in the rabbit, but that there is even more blood; and his observations upon bitches, cats, and other mammalia were equally confirmatory.

At p. 266, Pouchet says :—"In a work on the physical and moral system of woman, Roussel (*Système Physique et Moral de la Femme*, Paris, 1813, also edited by Cerise, 1860, Paris) pretends that menstruation is due to civilization. We have expressed nearly the same opinion; only we think that the social state has not determined the essence of the phenomenon, but that it has only considerably augmented its frequency in rendering it nearly mensual."

Auber also attributes the existence of the function to social advancement (Raciborski, p. 18). "Velpeau (*Tr. Comp. de l'Art des Accouch.*, t. I, p. 126) says that, in Lapland and Greenland, women are not more often regular than every three months ; and Gardien (*Tr. d'Accouch. et de Mal. des Femmes*, t. I, p. 233) pretends that in women in Polar countries the menstrual flow takes place only twice or thrice a year." Pouchet, therefore, ably argues the physiological identity of the function in all the mammalian series, including women ; he says (p. 227) :—"Menstruation consists in the appearance of a periodic and temporary excitement in the genital apparatus of woman. This function is declared by an afflux of blood in all the organs that share in it, and by the flow externally of a certain quantity of this fluid. Then it is essentially and ordinarily characterized by a swelling and maturation of one of the Graafian vesicles, and by the emission of the ovule which the latter contains."

(*To be continued.*)

